# Yes-Associated Protein in Atherosclerosis and Related Complications: A Potential Therapeutic Target That Requires Further Exploration

**DOI:** 10.3389/fcvm.2021.704208

**Published:** 2021-08-27

**Authors:** Congrui Sun, Bin He, Mingsheng Sun, Xiaoshuo Lv, Feng Wang, Jie Chen, Jianbin Zhang, Zhidong Ye, Jianyan Wen, Peng Liu

**Affiliations:** ^1^Peking University China-Japan Friendship School of Clinical Medicine, Beijing, China; ^2^Department of Cardiovascular Surgery, China-Japan Friendship Hospital, Beijing, China

**Keywords:** atherosclerosis, Yes-associated protein, Hippo pathway, intraplaque hemorrhage, vascular calcification

## Abstract

Atherosclerosis and its complications diseases remain leading causes of cardiovascular morbidity and mortality, bringing a massive burden on public health worldwide. Atherosclerosis is recognized as chronic inflammation, and involves several highly correlated processes, including lipid metabolism dysfunction, endothelial cell dysfunction, inflammation, oxidative stress, vascular smooth muscle cell activation, platelet activation, thrombosis, altered matrix metabolism, and vascular remodeling. Within the past few decades, accumulating evidence has shown that the Yes-associated protein (YAP), the major effector of the Hippo pathway, can play a crucial role in pathogenesis and development of atherosclerosis. Activation of YAP-related pathways, which are induced by alerting flow pattern and matrix stiffness among others, can regulate processes including vascular endothelial cell dysfunction, monocyte infiltration, and smooth muscle cell migration, which contribute to atherosclerotic lesion formation. Further, YAP potentially modulates atherosclerotic complications such as vascular calcification and intraplaque hemorrhage, which require further investigation. Here, we summarized the relevant literature to outline current findings detailing the relationship between of YAP and atherosclerosis and highlight areas for future research.

## Introduction

Atherosclerosis, a pathologic process underlying most cerebrovascular and cardiovascular diseases such as ischemic heart disease and stroke, remains a predominant cause of morbidity and mortality globally, carrying a considerable burden on public health (1, 2). Atherosclerosis is characterized by chronic inflammation in the arterial wall and, is triggered by endothelial cell dysfunction and structural alterations, including loss of the continuous luminal elastin layer and the exposure of proteoglycans (3). This process, impairs the endothelial barrier and permits the subendothelial aggregation of low-density lipoprotein (LDL) to form asymmetric focal thickenings of the intima. Endothelial cells can be activated by oxidized LDL (ox-LDL) and other risk factors that promote expression of adhesion proteins and release of chemokines, which prompts intimal immune cell infiltration and deposition of platelet-derived chemokines. Fatty streaks, the early lesions that consist of T cells and macrophage-derived foam cells, are prevalent in young people and, may progress to atheroma or eventually disappear. Continuous aggregation of cellular debris and cholesterol crystals can form the necrotic core of the plaque. Fibrous plaques can be covered by a fibrous cap composed of collagen fiber and smooth muscle cells, which can be infiltrated by macrophages in the thinning inflamed caps that are prone to rupture (4). During plaque formation, various histological alterations (e.g., calcification, intraplaque hemorrhage) can occur in the involved artery, which may contribute to the plaque vulnerability.

Atherosclerotic lesions occur primarily in arterial branches, bifurcations, and curvatures, with uneven distribution of lesions throughout the vascular tree (5). This site-specificity gives rise to the hydromechanics effect on the pathogenesis of atherosclerosis, where blood flow-induced shear stress plays a vital role in determining where most vascular lesions originate. The correlation between shear stress, produced by different flow patterns, and atherosclerosis has been investigated since its first introduction by Caro et al. in 1969 (6). Multiple studies revealed that it was the vascular endothelium has different behavioral responses to flow patterns both at the cellular and molecular levels, mediating the hydromechanics effect. The molecular mechanisms underlying endothelial cells mechanotransduction by which cells translate these physical and mechanical cues into biochemical signals, thereby controlling activation and phenotypic changes include multiple mechanosensitive transcription factors, such as NF-κB,HIF-1α, KLF2/4, AP-1, and NRF2 (7). Additionally, Yes-associated protein (YAP) is involved in the pathogenesis of atherosclerosis as a critical mediator in mechanosensing and signal transduction of endothelial cells.

Initially identified as a protein that interacts with the Src family tyrosine kinase Yes protein (8), YAP is a transcription cofactor that can act both as a corepressor and a coactivator and is a critical downstream regulatory target in the Hippo signaling pathway crucial to cell mechanotransduction processes (9). In 2014, Chitragari et al. first reported the relationship between flow pattern and YAP, where altered flow pattern stimulates changes in YAP expression in endothelial cells (10). This concept was further studied by Wang et al. in 2016, whose study showed that YAP and coactivator TAZ mediate flow-dependent endothelial phenotypes switching to modulate atheroma formation (11). Apart from mediating hydromechanics effects, over the past few years, mounting evidence also has demonstrated YAP activity in other pathophysiological processes leading to initiation and progression of atherosclerotic lesions, such as monocyte infiltration, smooth muscle cell activation, and *trans*-differentiation. The emerging roles of YAP makes it a potential target to develop prevention and treatment strategies for atherosclerosis. Therefore, we review current findings to elucidate the role of YAP in atherosclerosis and related complications.

## YAP and its Regulation

### Basic Structure of YAP

**Figure 1:**
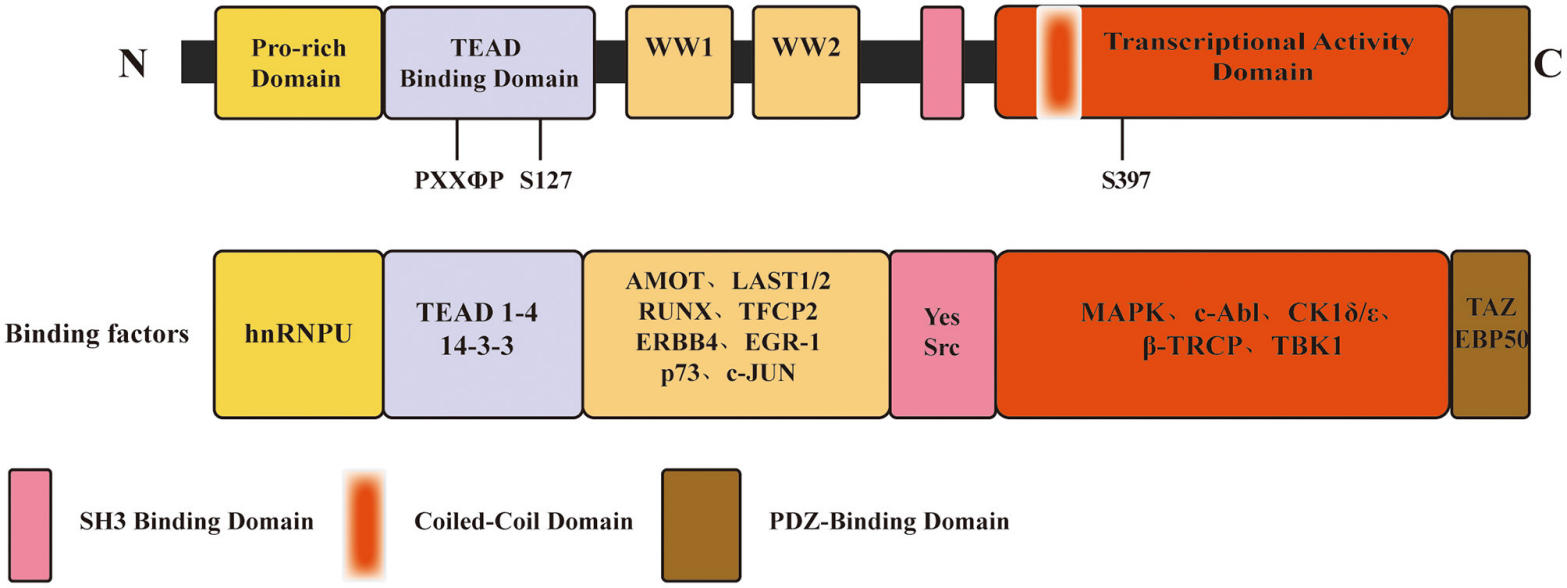
Schematic of YAP domain architecture, major binding sites and factors. From N-terminus to C-terminus, YAP contains a proline-rich domain, a TEAD-binding domain, one or two WW domains, an SH3-binding domain, a coiled-coil domain, a transcription activation domain, and a C-terminal PDZ-binding domain. The **PXXΦP** motif in the TEAD binding domain is important for the interaction between YAP and TEAD 1-4. **S127, S397**, primary phosphorylation sites by LATS1/2, which is a major modulation of YAP function.

YAP (also termed as YAP65 or YAP1) is a 65 kDa, proline-rich phosphoprotein. It was first identified in 1994 by Sudol et al. due to its binding to the SH3 domain of the Src family of non-receptor tyrosine kinase Yes, from which it got its name (8). According to the human genome database (*http://www.ncbi.nlm.nih.gov)*, the coding gene named Yes1 associated transcriptional regulator (*YAP1*) has nine transcripts encoding nine YAP isoforms. From N-terminus to C-terminus, YAP contains a proline-rich domain, a transcriptional enhancer associate domain (TEAD)-binding domain, one or two WW domains, an SH3-binding domain, a coiled-coil domain, a transcription activation domain, and a C-terminal PDZ-binding domain (12). YAP isoforms mainly differ in the number of WW domains and in the amino acids sequence in the TEAD-binding domain (13). In Figure 1, we have illustrated the YAP domain structure and several associated binding factors. The WW domain, composed of two strictly conserved tryptophan residues, refers to a protein-protein interaction domain and recognizes the PPxY (proline/proline/any amino acid/tyrosine) motif contained in various protein factors (14, 15). These protein factors include transcription factors and regulatory proteins that interact with YAP, such as large tumor suppressor (LATS) (15), runt-related transcription factor (RUNX) (16), activating protein-2 (AP-2) (17), angiomotins (AMOTs) (18), c-Jun, and C/EBPα (19). Whether YAP bears one or two WW domains is dependent on alternative splicing, classifying YAP into two isoforms: YAP1-1 (with one WW domain) and YAP1-2 (with two WW domains) (13). As a transcriptional cofactor, it lacks the DNA-binding domain, which requires interaction with other cofactors or transcriptional factors through the structural binding site to perform transcriptional regulation. Biological activity of YAP is mainly mediated by forming a complex with the TEAD transcription factors family members 1–4. A highly conserved loop called the PXXΦP (proline/ any amino acid/ any amino acid/ hydrophobic residue/ proline) motif in the N-terminal TEAD-binding domain is crucial to the interaction between YAP and TEAD factors (12).

### Regulation of YAP

**Figure 2:**
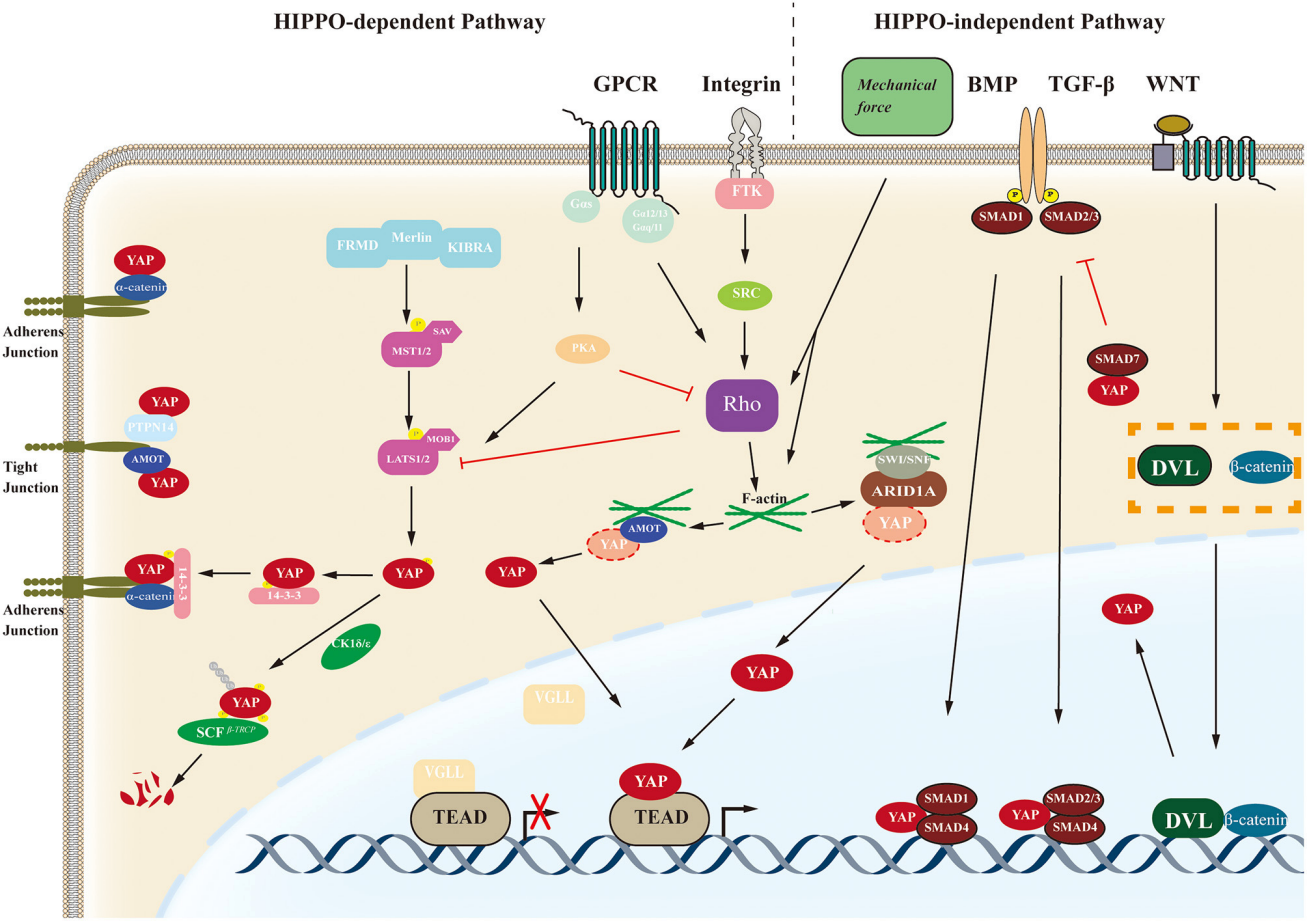
Schematic of the YAP regulatory pathway. The biological function of YAP are regulated by the Hippo-dependent pathways (including the Hippo pathway and other cross-talk signal pathways such as GPCR-mediated pathways and Integrin-mediated pathways) and Hippo-independent pathways such as BMP, TGF-β, WNT, etc. YAP, yes-associated protein; GPCR, G protein-coupled receptors; BMP, bone morphogenetic protein; AMOT, angiomotin; FRMD, FERM domain-containing protein; MST1/2, Mammalian STE20-like protein kinase 1/2; SAV, Salvador family WW domain-containing protein; LAST1/2, Large tumor suppressor 1/2; MOB1, MOB kinase activator 1; FTK, fusion tyrosine kinases; TEAD, transcriptional enhancer associate domain; ARID1A, AT-rich interactive domain-containing protein 1A; SWI/SNF, switch/sucrose non-fermentable complex.

YAP transcriptional activity depends on its subcellular location, which is regulated by multiple signaling pathways through which YAP can exert versatile effects on various biological processes such as cell proliferation, cell fate determination, tumorigenesis and mechanosensing. The regulatory signal pathways of YAP are illustrated in Figure 2.

#### Hippo Pathway

Amongst those pathways associated with YAP, the Hippo pathway is the most classical. The Hippo pathway was first discovered in *Drosophila* and is highly conserved. The pathway in mammals mainly consists of mammalian STE20-like protein kinase 1/2 (MST1/2), large tumor suppressor 1/2 (LATS1/2), Salvador family WW domain-containing protein 1 (SAV1), MOB kinase activator 1A/B (MOB1), mitogen-activated protein 4 kinase 4, YAP, transcriptional co-activator with PDZ-binding motif (TAZ), and its downstream target TEAD family members 1–4 (15, 20–23). TAZ, homologous to YAP, has similar molecular architectures and biological functions, often referred to as YAP/TAZ (24). The Hippo pathway mainly suppresses tumor oncogenesis in the physiological state, and activation can be induced *via* multiple stimuli. After being activated by upstream stimuli, the pathway is initiated by phosphorylation of MST1/2 associated with SAV1, which can further stimulate LATS1/2 and its cofactor MOB1 by phosphorylation. Phosphorylation of the complex further phosphorylates the transcription cofactors YAP, in turn, leading to YAP binding with 14-3-3 protein in the cytoplasm and inhibiting nuclear translocation of YAP. While the Hippo pathway is inactivated, the unphosphorylated YAP translocates to the nucleus and binds to the TEAD transcription factor family to regulate the expression of target genes including MYC proto-oncogene bHLH transcription factor (*MYC*), baculoviral IAP repeat-containing 5 (*BIRC5*), AXL receptor tyrosine kinase (*AXL*), connective tissue growth factor (*CTGF*), or cysteine rich angiogenic inducer 61 (*CYR61*). Moreover, the stability of YAP can be modulated through its phosphorylation by the casein kinase 1 ε/δ (CK1 ε/δ) complex, further inducing its ubiquitination by the recruitment of the E3 ubiquitin ligase Skp1-Cul1-F-box β-transducin repeat-containing protein (SCF-β-TrCP). The process above ultimately leads to degradation of YAP by the proteasome (25).

#### Non-Hippo Pathways

Other signaling pathways, such as the G protein-coupled receptor (GPCR), Wnt, transforming growth factor-β (TGFβ), epidermal growth factor (EGF), and Notch pathways, also have regulatory effects on YAP transcriptional activity through crosstalk with the Hippo pathway or independent of the Hippo pathway (26–30). Moreover, YAP/TAZ can also be regulated by mechanical signals from cell microenvironment (9, 31, 32). In fact, YAP/TAZ plays crucial roles in cellular mechanosensing, affecting differentiation direction and cell fate. Extracellular matrix rigidity and cell geometry regulate YAP/TAZ transcriptional activity through Rho GTPase activity and tension of the actomyosin, independent of the Hippo cascade (9). High extracellular matrix rigidity and wide cell spreading upshift YAP/TAZ nuclear location. Chang et al. reported a completely different mechanism in 2018, showing that the switch/sucrose non-fermentable (SWI/SNF) chromatin-remodeling complexes competitively inhibit interaction between YAP/TAZ and TEAD factors by binding with YAP/TAZ *via* mediation of the AT-rich interactive domain-containing protein 1A (ARID1A) subunit. This kind of modulation of YAP activity can be regulated by cellular mechanical signals under low mechanical stimulation, the SWI/SNF–YAP/TAZ complex forms, while under conditions of high mechanical stress, nuclear F-actin fibers bond with SWI/SNF complexes, which promotes formation of YAP/TAZ-TEAD complexes to exert transcriptional activities (33).

#### Post-translational Modifications

Some PTMs on various amino acid residues, including phosphorylation, *O*-GlcNAcylation, acetylation, methylation, and ubiquitination, also can regulate YAP transcriptional activity, some signal pathways mentioned above also have modulating effects. Phosphorylation is the primary post-translational regulator of YAP activity. In the context of the Hippo pathway, YAP is phosphorylated by LATS1/2 at five serine residues (Ser61, Ser109, Ser127, Ser164, and Ser397), two of which are major sites involved in regulating YAP activity (34). Phosphorylation of the Ser127 site by LATS1/2 leads to interaction with 14-3-3 protein, causing cytoplasmic retention and activity inhibition of YAP. Further, the nuclear Dbf2-related kinases (NDR1/2) also can phosphorylate Ser127 of YAP, leading to cytoplasmic retention and promotion of apoptosis and cell cycle regulation (35). LATS1/2 phosphorylation of Ser397 regulates protein stability by facilitating further phosphorylation of YAP on Ser400 and Ser403 by CK1ε/δ, leading to a “phosphodegron motif” formed with pS397/pS311 in the C-terminal region of YAP. This motif can recognize and recruit an adaptor of SCF E3 ubiquitin ligase called β-TrCP, which targets YAP for ubiquitination and facilitates proteasomal degradation (36). JUN N-terminal kinases (JNK1 and JNK2), in addition to the Hippo pathway, can phosphorylate YAP at five sites (Thr119, Ser138, Thr154, Ser317, and Thr362), leading to various effects dependent on cell context (37). The non-receptor tyrosine kinase c-Abl is reported to phosphorylate YAP at Tyr357 to promote the binding affinity of YAP and p73, leading to proapoptotic gene expression (38). However, another non-receptor tyrosine kinase Yes phosphorylates YAP on the same site with c-Abl to induce an opposite effect. The phosphorylation promotes formation of the β-catenin–YAP complex, leading to YAP inhibition and downregulation of gene expression (39). The Ser/Thr protein kinase cyclin-dependent protein kinase 1 (CDK1) phosphorylates YAP at three residues (Thr119, Ser289, and Ser367), to regulate the cell cycle and promote cell migration and invasion (40). However, evidence indicates another three residues where CDK phosphorylates YAP: Ser138, Thr143, and Ser367 (41).

Apart from phosphorylation, *O*-GlcNAcylation is another PTM that alters YAP function, in which O-linked b-N-acetylglucosamine (*O*-GlcNAc) modifications are attached to serine or threonine residues by O-GlcNAc transferase (42). Zhang et al. found that *O*-GlcNAc transferase acts at the Thr241 site on YAP to promote YAP stabilization and target gene expression (43). At Ser109, *O*-GlcNAc transferase acts on YAP to disrupt the interaction between YAP and LAST1 (44). Other PTMs that have a regulatory effect on YAP include acetylation, methylation, and ubiquitination. The key enzymes and acting sites involved in these PTMs are summarized in Table 1 with the sequential effects.

**Table 1:** Summary of major post-translational modifications (PTM) patterns of YAP.

**Modification**	**Enzyme**	**Acting Site**	**Effect**	**References**
Phosphorylation	LATS1/2	Ser 127	Cytoplasmic retention by binding of 14-3-3	(45)
				
		Ser 397	Facilitates further serine site phosphorylations	
	CK1δ/ε	Ser 400 Ser 403	Generates a “phosphodegron” with Ser 397	(45)
	c-Abl	Tyr 357	Increasing the binding affinity of YAP1 and p73, activating proapoptotic gene expression	(38)
			Activate endothelial cell dysfunction	(46)
	Src/YES1		Induces formation of the β-catenin–YAP complex	(39)
	CDK1	Thr119, Ser289, Ser367	Regulating cell cycle	(40)
		Ser138, Thr143, Ser367		(41)
	NDR1/2	Ser127	Cytoplasmic retention by binding of 14-3-3	(35)
	JNK (JNK1 and JNK2)	Thr119 Ser138, Thr154 Ser317, Thr362	Inducing cell-context dependent effect of YAP	(37)
Ubiquitination	SCFβ-TRCP	/	Protein degradation	(36)
	Fbxw7			(47)
	TRAF6	Lys252	Upregulates chemokine production and macrophage migration	(48)
Monomethylation	Set7	Lys 494	Cytoplasmic retention	(49)
Acetylation	CBP/p300		Changing transcriptional activity	(50)
Methylation	SETD7	Lys494	Cytoplasmic localization	(49)
			YAP/β-catenin complex stabilization and β-catenin nuclear relocalization upon WNT stimulation	(51)
O-GlcNAcylation	O-GlcNAc transferase	Thr241	Stabilizes YAP and promotes oncogenic functions of YAP	(43)
		Ser109	Disrupts the interaction between YAP and LATS1	(44)

## YAP in the Initiation and Development of Atherosclerosis

### YAP and Endothelial Cell Dysfunction

Endothelial cells, lining the inner layer of the vasculature and located at the interface between the circulation and extracellular matrix of the vessel wall, respond to multiple environmental stimuli, mainly from blood flow, such as ox-LDL and shear stress, leading to endothelium inflammation and dysfunction which triggers the process of atherosclerosis. Among these stimuli, shear stress is responsible for the uneven distribution of atherosclerotic lesions throughout the arterial tree. Shear stress is produced by different blood flow patterns, including laminar flow and distributed flow, and can modulate endothelial phenotypes and intercellular junctions, playing crucial roles in endothelial dysfunction. YAP mediates this process as a mechanosensitive transcription factor, in which laminar flow (unidirectional flow) with high shear stress induces endothelial protective effects and disturbed flow with oscillatory wall shear stress evokes proatherogenic responses (5, 7).

In 2016, three research teams reported independently that flow-dependent transactivation of YAP in endothelial cells regulates initiation and development of atherosclerosis (11, 52, 53). By exposing cultured endothelial cells (including human umbilical vein endothelial cells (HUVEC) and human aortic endothelial cells) to disturbed flow, Wang et al. found that YAP was activated and translocated into the nucleus to increase the expression of target genes. These target genes, including *CYR61, CTGF*, and ankyrin repeat domain 1 (*ANKRD1*), prompted inflammation and proliferation in endothelial cells and were inhibited by laminar flow exposure through YAP inactivation. The authors further explored the effect of YAP inhibition on atherosclerotic lesion development in the partial-ligation *ApoE*^−/−^ mouse model and found that *in vivo* inhibition of YAP/TAZ activation significantly reduces the total burden of atherosclerotic plaques. These results indicated that disturbed flow induces YAP activation to promote the atheroprone phenotypes switch in endothelial cells and atherosclerosis development (52). However, the mechanism through which flow patterns act on YAP translocation remained unexplained.

Another study conducted by Xu et al. may provide a plausible answer that shear stress-regulated YAP/TAZ activity is dependent on the Hippo pathway. They discovered that laminar flow markedly increases YAP phosphorylation at Ser127 in a LATS1/2-dependent manner of in the HUVEC model, which leads to cytoplasmic retention and activity inhibition of YAP (53). This study, however, failed to provide further information about how the flow pattern influenced signaling the upstream of the Hippo pathway. More detailed research performed by Wang et al. demonstrated that overexpression of integrin β3 by cytoplasmic-domain-deleted integrin (β3Δcyto) reversed laminar flow-induced YAP phosphorylation in HUVECs, whereas knockdown of integrin β3 or G-protein subunit Gα13 attenuates YAP phosphorylation. Given that the physical interaction between integrin β3 and Gα13 induces RhoA inhibition, the study also found that overexpression of RhoA by transfecting HUVECs with constitutively active RhoA (Q63L) reduces YAP phosphorylation induced by laminar flow. Further experiments showed that activation of YAP increased JNK signaling to induce adhesion molecule expression and inflammation in endothelial cells. These results indicated that the atheroprone phenotype disturbs flow-induced endothelial YAP activation, which promotes inflammation and atherogenesis by enhancing JNK activity. By contrast, laminar flow exerts an atheroprotective effect by inhibiting YAP through the integrin β3/ Gα13/RhoA pathway (11).

Another integrin-related pathway is be involved in the regulation of YAP activity by disturbed flow. Li et al. found that oscillatory shear stress led to YAP phosphorylation at Y357 instead of Ser127 following integrin α5β1 activation, which eventually promotes nuclear translocation of YAP in endothelial cells. Further mechanistic studies showed that a non-receptor protein tyrosine phosphatase c-Abl mediates this process. Inhibition of c-Abl attenuates the integrin α5β1-induced YAP tyrosine phosphorylation. Thus, disturbed flow activates YAP transcriptional activity by phosphorylation at Y357 through the integrin α5β1/c-Abl pathway (46). Li et al. also found that specific knock-down of *YAP* in endothelial cells obviously retarded plaque formation compared with control group in carotid ligation model of *ApoE*^−/−^ mice. However, more detailed information about plaque morphology, stability indices and cell composition of YAP knock-down artery was not given in the article. Interestingly, integrin α5β1 also mediates another mode of YAP activity by fibronectin (54), a substance that can be induced by atherogenic shear stress and contributes significantly to inflammation signaling in the atheroprone site (55, 56). Yun et al. found that fibronectin bonded with integrin α5 and then recruited and activated phosphodiesterase 4D5 (PDE4D5) by inducing its dephosphorylation. Activated PDE4D5 then facilitated B55α-PP2A complex assembly, which promoted YAP dephosphorylation and activation, leading to endothelial inflammation and atherosclerosis initiation (54). This study provided a novel mechanism regulating flow-dependent YAP transactivation. Moreover, Yuan et al. conferred a novel insight into this issue. Their experiment showed that unidirectional flow (laminar flow) inhibits YAP activity by promoting autophagy and inducing SIRT1-mediated YAP deacetylation, which further facilitates nuclear export and subsequent degradation of YAP, while in the context of this experiment, disturbed flow had no effect on autophagy and YAP acetylation (57). The molecular mechanism underlying disturbed flow-dependent YAP nuclear translocation and activation requires further exploration.

Amongst cellular microenvironment, matrix stiffness is another mechanical cue that is highly associated with atherosclerotic burden and able to regulate YAP/TAZ activity. Carotid-femoral pulse wave velocity (cf-PWV), a gold standard for arterial stiffness measurement (58), has been proven to have positive correlation with atherosclerotic burden indicators such as carotid intima–media thickness and carotid plaque presence (59). Through atomic force microscopic study (ATM), Hayashi et al. have found in rabbit model endothelial cells in medial wall of aortic bifurcation, where the flow pattern is turbulent, are stiffer than other regions, indicating that matrix stiffness can be regulated by shear stress (60). In turn, matrix stiffness can also affect endothelial response to shear stress *via* YAP/TAZ Recently, a study performed on vessel-chip model revealed that substrate stiffness modulated the endothelial shear mechanoresponse (61). Stiffer substrates increased nuclear localization of YAP in endothelial even under high shear, whereas low shear strongly increased nuclear localization of YAP across stiffnesses. Combing with previous findings, this result indicates that high matrix stiffness play a casual role in initiation of atherosclerosis *via* activation of YAP/TAZ.

Moreover, YAP also is involved in many other atherogenic factors that mediated endothelial cells dysfunctions. Junctional protein associated with coronary artery disease (JCAD) is an intercellular junctional protein in endothelial cells that is, significantly associated with myocardial infarction and coronary artery disease (62, 63). JCAD regulates pathological angiogenesis, promotes endothelial cells proliferation, and inhibits apoptosis (62). Recent studies reveal that JCAD also can promote atherosclerosis by inducing endothelial cells dysfunction *via* YAP regulation (64, 65). Jones et al. found that JCAD activates YAP by interacting with LATS2 and inhibiting its activity, resulting in YAP activation and expression of target genes (e.g., *CTGF, CCND1, and BIRC5*). They also found that JCAD mediated Hippo/YAP pathway regulation in a RhoA-dependent manner (64). Given that RhoA is downstream of the integrin β3/ Gα13/RhoA pathway mentioned above (11), this result complements previous work and indicates that JCAD regulation can be flow-pattern dependent. Intriguingly, this concept was validated by another study showing that atheroprotective unidirectional laminar flow inhibited, while atheroprone disturbed flow enhanced, JCAD expression in human endothelial cells (65). Additionally, ox-LDL and cholesterol crystal treatment increase *JCAD* gene expression, adding an alternative explanation to ox-LDL regulation of YAP in the context of atherosclerosis. JCAD promotes YAP/TEAD formation by interacting with the actin-binding protein TRIOBP, leading to stabilization of F-actin stress fibers. Additionally, a cytosolic adaptor protein called ShcA promotes atherosclerotic progression by triggering nuclear translocation of YAP, which further prompts intercellular adhesion molecule (ICAM) expression and endothelial inflammation (66). ox-LDL, a potent atherogenic factor (67), induces endothelial cells dysfunction through suppression of YAP rather than activation (68–70). Hu et al. showed that ox-LDL induces endothelial cells dysfunction and atherosclerosis by increasing miR-496 expression, followed by reducing YAP protein expression (68). Vestigial-like 4 (VGLL4) and naringin protect endothelial cells from apoptosis and inflammation by reversing ox-LDL-induced YAP attenuation (69, 70). Given that endothelial cell-specific knockdown of *YAP1* gene attenuates endothelial cells inflammation *in vitro* and atherosclerotic plaque burden *in vivo* (11, 46, 52), it stands to reason that these contradictory results mentioned above still have doubts in credibility due to the lack of information about YAP subcellular location protein and need further exploration and validation. Different cell types and inflammatory stimuli may be possible causes of these discrepancies.

### YAP and Monocyte Infiltration and Macrophage Activation

**Figure 3:**
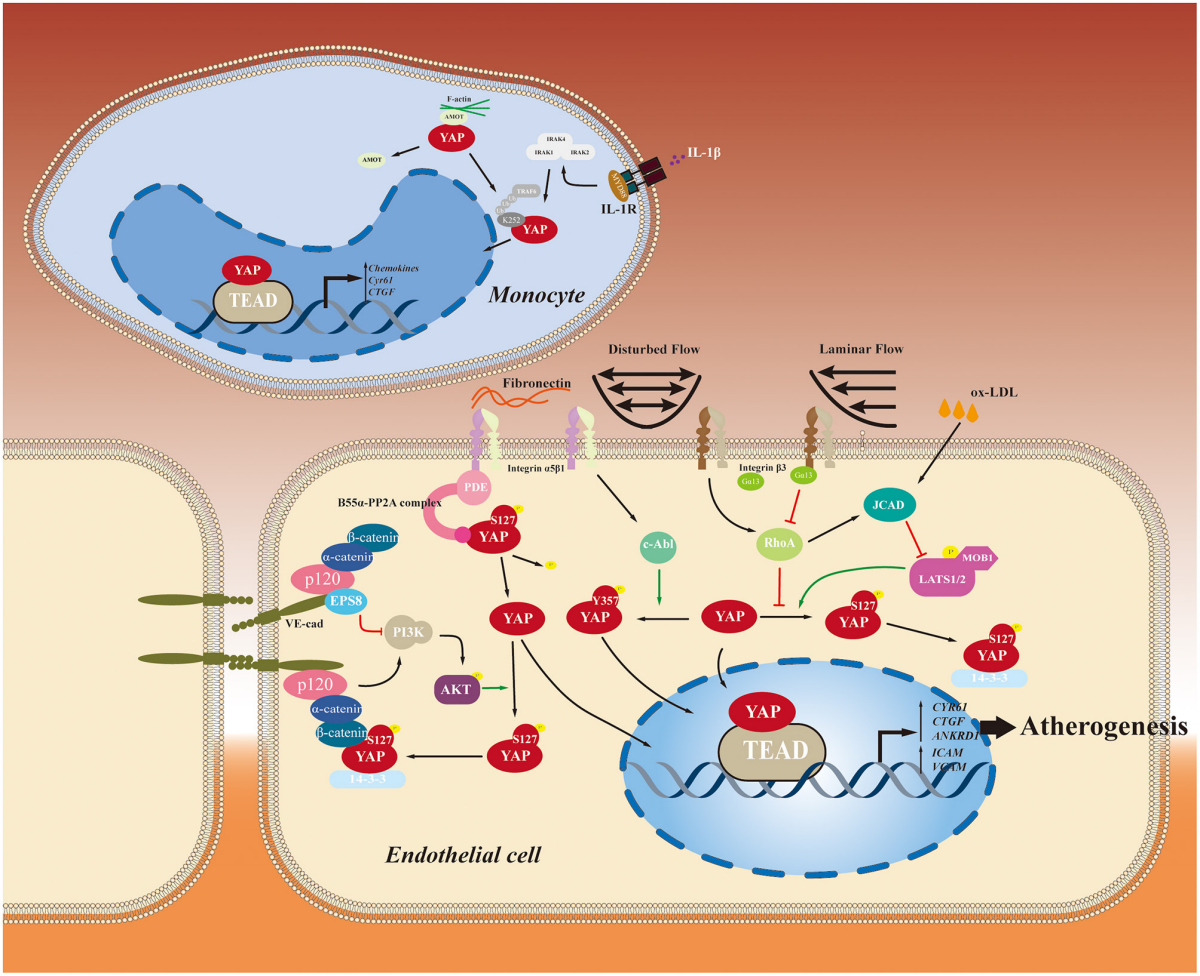
YAP roles played in endothelial cells and monocytes in the context of atherosclerosis initiation. YAP-related pathways involved in the initiation and development of atherosclerosis are summarized.

During atherosclerosis development, activated endothelial cells recruit blood monocytes to migrate into the intima. Then monocytes differentiate into macrophages that absorb lipid deposits and cellular debris to form foam cells, further accumulating to form a lipid pool (71). By specific YAP/TAZ knockdown in monocytes, Wang et al. (52) found that the up-regulation of β2 integrin and *ICAM1* induced by tumor necrosis factor-α (TNFα) in THP1 cells is blunted, leading to inhibition of their adherence to the activated endothelial cells without altering the plasticity of THP1 monocyte-to-macrophage differentiation (52). Moreover, YAP activation in macrophages by TNF receptor-associated factor 6 (TRAF6)-mediated YAP ubiquitination at Lys252 upregulates chemokine production and macrophage migration, accelerating atherosclerotic lesions formation (48). Macrophage phenotype switching from M1 to M2 subtypes plays a crucial role in atherosclerotic inflammation recession and plaque regression (72). In chronic inflammatory disease that YAP impairs the interleukin-4 (IL-4)/IL-13-induced M2 macrophage polarization, whereas YAP promotes the lipopolysaccharide/interferon γ -triggered M1 macrophage activation (73). However, it remains unclear how YAP regulates macrophage polarization in atherosclerosis. Thus, further study on this aspect is needed in the future. YAP roles played in endothelial cells and monocytes in the context of atherosclerosis initiation are summarized in Figure 3.

### YAP and Vascular Smooth Muscle Cell Proliferation and Migration

In response to multiple atherogenic factors, vascular smooth muscle cells (VSMCs) undergo a phenotype switch from contractile to synthetic, migrate to the intima from the media, and proliferate, leading to atherosclerotic plaque development (74, 75). VSMCs also can transdifferentiate into macrophage-like cells in the plaque (76). YAP also is associated with VSMC migration and proliferation. Although study has shown that specific *YAP1* knockout in VSMCs in mice results in abnormal development of large arteries and perinatal lethality (77), there is a lack of studies performing SMC-specific knock-down of YAP in atherosclerosis models. Kimura et al. reported that Hippo pathway activation promotes cAMP-induced actin-cytoskeleton remodeling to inhibit YAP/TEAD complex-dependent expression of pro-mitogenic genes, leading to proliferation inhibition (78). A Kunitz-type proteinase inhibitor called tissue factor pathway inhibitor-1 (TFPI-1) is a major physiological inhibitor of the TF-initiated coagulation process. Xiao et al. found that *TFPI-1* knockout in VSMCs reduces interaction between TFPI-1 and AMOT, which led to a decrease in YAP phosphorylation, thus increasing nuclear translocation of YAP. Further, this induces VSMC migration and proliferation, contributing to atherosclerosis (79). Above all, these results indicate that activation of YAP can promote VSMC proliferation and migration which contribute to atherosclerotic plaque development. However, VSMC can also generate extracellular matrix to form the fibrous cap and hence stabilize plaques in late-stage lesion (69). There is a lack of studies on YAP' activity in VSMC of late-stage lesions which need further exploration.

## YAP in Atherosclerotic Complications

### YAP and Atherosclerotic Calcification

Arteriosclerotic calcification, commonly regarded as a late-stage complication of atherosclerotic plaques, is an active process under tight regulations and is an independent predictor of cardiovascular morbidity and mortality (80). Although calcification of atherosclerosis plaques used to be considered unregulated and a passive deposition of calcium in the arterial wall, it is now well-recognized as a complex pathophysiological phenomenon resembling intramembranous ossification (81). YAP can interact with various factors involved in osteoblast development and maturation, such as Runx2 (82), bone morphogenic protein-2 (BMP-2) (83), and β-catenin (84). YAP plays a crucial role in human adipose-osteogenic differentiation, where increasing YAP activity, either pharmacologically or by genetic manipulation, enhances osteogenic differentiation but suppresses differentiation to adipocytes (85). VSMCs undergo *trans*-differentiation and osteogenic differentiation and make a significant contribution to vascular calcification (86). A recent study showed that cytoplasmic YAP/TAZ in VSMCs interacts with disheveled 3 (DVL3) to avoid its nuclear translocation, thereby inhibiting osteogenic differentiation (87). In high phosphate-induced vascular calcification models, increasing YAP activity through glycosylation by *O*-GlcNAc transferase promotes VSMC osteogenesis and vascular calcification (88). However, the network regulating YAP activity in atherosclerotic calcification still requires further research.

### YAP and Intraplaque Hemorrhage

Intraplaque hemorrhage is a feature of advanced atherosclerotic plaques and is considered a critical factor in plaque growth and vulnerability (89). Angiogenesis in the vasculature is a significant cause of intraplaque hemorrhages (90). Recently, several studies have discovered that YAP plays a critical role during angiogenesis. Activation of YAP, induced by multiple stimuli, such as knockdown of the E3 ligase *HECW2*, vascular endothelial growth factor (*VEGF*), and Cellular communication network factor 1 (*CCN1*), upregulates *ANG-2* expression, thereby promoting angiogenic sprouting (91–93). These results indicate that YAP has a potential role in intraplaque hemorrhage by promoting angiogenesis, although further research is needed to elucidate a direct linkage.

## YAP As the Target of Anti-Atherosclerosis Therapeutic Strategies

### Verteporfin

Verteporfin (VP) is a second-generation lipophilic photosensitizer derived from benzoporphyrin and, contains an aromatic heterocyclic ring molecule and four modified pyrrole units interconnected through methine bridges. It is used clinically in photodynamic therapy to treat neovascular macular degeneration (94, 95). VP is a potent YAP inhibitor and disrupts physical interactions between YAP and TEAD (96). In a study performed in an *ApoE*^−/−^ mouse model by Jain et al., intra-arterial administration of VP caused accumulated of VP in atherosclerotic plaques in a short time inducing plaque macrophages apoptosis which may inhibit plaque development. However, evidence is still lacking regarding the direct effect of VP on the size of atherosclerotic plaques. Additionally, further detailed research required on the efficacy and safety of photodynamic therapy on atherosclerosis using VP.

### Statins

As the most commonly used anti-atherosclerosis drug, statins inhibit YAP activation and prevent YAP-mediated tumor growth; this effect is, regarded as one of the pleiotropic effects of statins (97, 98). Wang et al. provided an alternative explanation for the pleiotropic effect where simvastatin inhibition of endothelial proliferation and inflammation is mediated by suppression of YAP activities. Simvastatin also can reverse YAP activation induced by disturbed flow (52). By performing a YAP reporter gene activity assay of various common anti-atherosclerotic agents, Wang et al. showed that statins produced the most substantial inhibitory effect on YAP activity (11).

### Methotrexate

Methotrexate (MTX) has beneficial effects in cardiovascular disease treatment. Long-term low dose MTX treatment reduced cardiovascular disease and cardiovascular mortality in rheumatoid arthritis patients (99). A recent study found that MTX inhibited disturbed flow-induced atherosclerosis lesion formation by attenuating inflammation processes in endothelial cells (100). Mechanism experiments showed that MTX represses disturbed flow-induced endothelial YAP and TAZ activation in an AMP-dependent, kinase-dependent manner. These results indicate that MTX exhibits an atheroprotective effect by inactivating YAP and TAZ *via* AMPK inhibition, leading to a reduction of endothelial cells inflammation.

### Harmine

Harmine is a kind of β-carboline alkaloid complex extracted from *Peganum. harmala*, a traditional Chinese medicinal plant that has been used to treat cardiovascular diseases (101). Yang et al. found that harmine had a potent atheroprotective role, inhibiting atherogenesis in both *ApoE*^−/−^ and *LDLR*^−/−^ mice by attenuating endothelial inflammation. Harmine inhibits disturbed flow-induced YAP nuclear translocation and endothelial cell activation by reducing phosphorylation of YAP at Tyr357. Further study showed harmine acted on YAP through protein tyrosine phosphatase non-receptor type 14 (PTPN14). These results indicated that harmine inhibits disturbed flow-induced endothelial cells activation *via* a PTPN14/YAPY357 pathway (102).

### Manganese Chloride

Manganese chloride (MnCl_2_) has anti-atherosclerotic effects in animal model (103). Oral administration of MnCl_2_ has been confirmed by Wang et al. to decrease atherosclerotic plaque formation by inhibiting YAP activation *via* activation of integrin in high-fat diet-fed ApoE^−/−^ mice (11). Its anti-atherosclerotic effect was also validated by Zhang et al. (104) recently. However, it still needs safety and effectiveness study performed on human body.

### Salvianolic Acid B

Salvianolic acid B (Sal -B) is an active constituent extracted from the root and rhizome of *Salvia miltiorrhiza*, a traditional Chinese herb beneficial to the cardiovascular system. Yang et al. provided evidence showing that Sal-B could protect endothelial cells and pericytes from inflammation, oxidative stress, and apoptosis to delay the atherogenesis. Further mechanistic studies showed that Sal-B inhibits of ox-LDL production possesses and anti-inflammatory functions by regulating the YAP/TAZ/JNK pathway. These results suggest that Sal-B inhibited YAP/TAZ activation and the subsequent JNK cascade to protect endothelial cells from inflammation *in vitro* and slow atherosclerosis development *in vivo* (105).

### Naringin

Naringin is a major compound extracted from citrus fruits such as grapefruits and tomatoes and has potential protective effects against atherosclerosis (106, 107). Zhao et al. revealed that Naringin protected endothelial cells from ox-LDL -induced cell injury and apoptosis, restored endothelial barrier integrity by preventing VE-cadherin disassembly and F-actin remodeling, and inhibited expression of pro-inflammatory factors including IL-1β, IL-6, and IL-18 in the HUVEC model. Mechanistic examination showed that naringin restored YAP inhibition induced by ox-LDL, indicating that naringin can promote YAP activity to reverse endothelial cell apoptosis, endothelial–mesenchymal transition and inflammation induced by ox-LDL (69).

### Scutellarin

Scutellarin is the main active flavonoid component extracted from breviscapine, has antioxidant, and anti-inflammatory effects, and is capable of scavenging oxygen-free radicals (108, 109). Fu et al. showed that Scutellarin inhibited phosphorylation of MST1, YAP, and forkhead box O3A (FOXO3A) to enhance nuclear translocation of FOXO3A, leading to the subsequent suppression of angiotensin II (AngII) induced apoptosis in the human aortic endothelial cells model and atherosclerotic burden in a rat model (110).

## Conclusion and Future Perspectives

YAP is a crucial transcriptional cofactor that is regulated by multiple stimuli and, mediates various pathways to initiate atherosclerosis development. The function of YAP is characterized by high cell-type dependence. Most of these researches, however, are performed on animal model, unanswered questions still remain regarding how in the regulation of YAP impacts atherosclerosis in human, requiring further exploration.

Furthermore, the crucial role of YAP in osteogenesis and angiogenesis can link it to the atherosclerosis complications which adds to the vulnerability of plaques such as vascular calcification and intraplaque hemorrhage. Recently a study conducted by our lab showed that the YAP expression is increased in symptomatic carotid plaques compared to non-symptomatic plaques, and these trends are mainly distributed close to angiogenesis and calcification areas, indicating the potential role of YAP in plaque stability (111). Hence, a hypothesis can be raised that YAP may have a role in determining plaque vulnerability for its involvement in angiogenesis and vascular calcification. This hypothesis requires further investigation in future studies.

## Author Contributions

CS wrote the first draft, which was revised by the remaining authors. All authors made a substantial contribution to conceptualization and writing and agreed to be accountable for the content of this review.

## Conflict of Interest

The authors declare that the research was conducted in the absence of any commercial or financial relationships that could be construed as a potential conflict of interest.

## Publisher's Note

All claims expressed in this article are solely those of the authors and do not necessarily represent those of their affiliated organizations, or those of the publisher, the editors and the reviewers. Any product that may be evaluated in this article, or claim that may be made by its manufacturer, is not guaranteed or endorsed by the publisher.
